# Epigenetic mechanisms involved in methamphetamine addiction

**DOI:** 10.3389/fphar.2022.984997

**Published:** 2022-08-26

**Authors:** Hang Wang, Xianghuan Dong, Maher Un Nisa Awan, Jie Bai

**Affiliations:** Laboratory of Molecular Neurobiology, Medical School, Kunming University of Science and Technology, Kunming, China

**Keywords:** methamphetamine, addiction, epigenetics, neurotransmitters, synaptic plasticity

## Abstract

Methamphetamine (METH) is an illicit psychostimulant that is widely abused. The molecular mechanism of METH addiction is complicated and still unknown. METH causes the release of the neurotransmitters including dopamine, glutamate, norepinephrine and serotonin, which activate various brain areas in the central nervous system. METH also induces synaptic plasticity and pathological memory enhancement. Epigenetics plays the important roles in regulating METH addiction. This review will briefly summarize the studies on epigenetics involved in METH addiction.

## Introduction

Methamphetamine (METH) is a psychostimulant that induces euphoria. METH has been widely abused in recent decades due to its high euphoric activity. The United Nations Office on Drugs and Crime (UNODC) reported that 27 million people used amphetamines worldwide, suggesting a significant increase of METH trafficking in the world (United Nations Office on Drugs and Crime, 2021).

**FIGURE 1:**
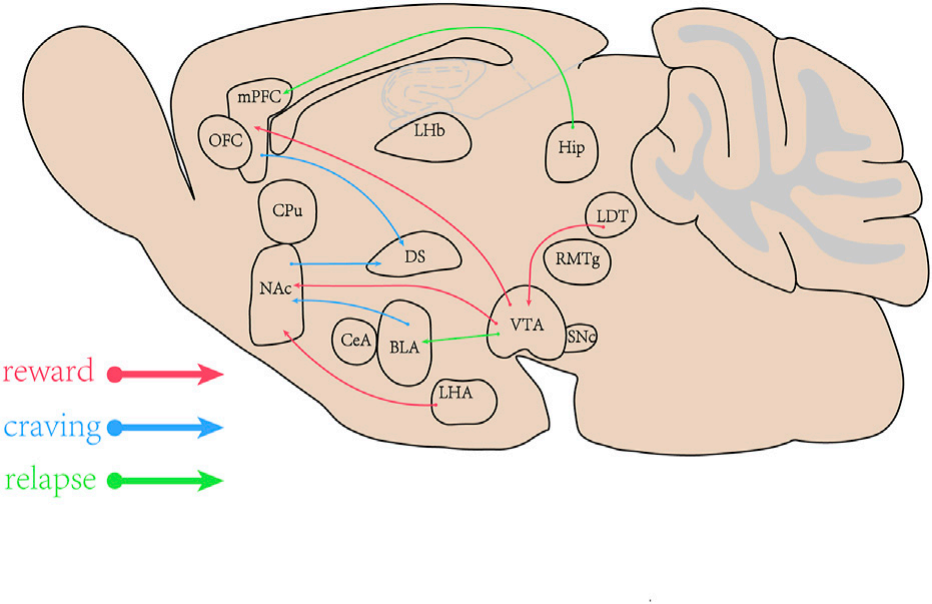
The brain areas involved in methamphetamine (METH) addiction. The ventral tegmental area (VTA), the nucleus accumbens (NAc), the dorsal striatum (DS), the medial prefrontal cortex (mPFC), the *Hippocampus* (Hip), amygdala (BLA) and their projects or inputs play the important roles in rewarding effects, craving and relapse induced by abuse drugs.

Substance abuse is a psychiatric disorder with the initiation of drug use and the development of uncontrolled drug intake, which includes rewarding effects, dependence, behavioral sensitization, drug craving and relapse. Major brain areas involved in the substance use are the ventral tegmental area (VTA), the nucleus accumbens (NAc), the dorsal striatum (DS), the medial prefrontal cortex (mPFC), the hippocampus (Hip), the basolateral amygdala (BLA), the central amygdala (CeA), the orbital-frontal cortex (OFC), the caudate putamen (CPu), the thalamus, the lateral hypothalamus (LHA), the substantia nigra pars compacta (SNc), the rostromedial tegmental nucleus (RMTg), the laterodorsal tegmentum (LDT), the dorsal raphe nucleus (DRN) and the lateral habenula (LHb) (Hu, 2016). The mesolimbic circuitry is composed of dopaminergic neurons in the midbrain VTA and their innervation of medium spiny neurons (MSNs) within the NAc and project into the mPFC (Hyman et al., 2006; Josselyn and Tonegawa, 2020). The NAc receives neuronal inputs from the VTA, the Hip, the BLA, and the thalamus. The DS receives neuronal inputs from the prefrontal cortex (PFC) and the NAc, and plays the important roles in regulating addiction. The mPFC regulates decision making, memory retrieval, learning cognitive functioning, and intense emotional responses. The areas involved in addiction are concluded in Figure 1. Substance alters the release of neurotransmitters including dopamine (DA), norepinephrine (NE), serotonin, glutamate (Glu) and γ-Aminobutyric Acid (GABA) throughout the brain (Smiley and Wood, 2022). The enhanced synaptic plasticity is related to memory and engrams in brain regions (Avchalumov et al., 2020). The synaptic plasticity induced by METH via D1 Rs results in modification of corticostriatal circuits and is involved in METH self-administration and addiction-like behavior (Gonzalez et al., 2019).

## Epigenetics

The epigenetics is molecular modifications to gene expression, but the DNA sequence is not changed. It can regulate DNA-related processes, such as DNA repair, chromatin organization, and RNA transcription and splicing which are inherited. Epigenetic events include histone modifications, chromatin remodeling, noncoding RNA, DNA methylation and others.

## Epigenetics and methamphetamine addiction

It is widely reported that epigenetic alterations are related to aberrant cellular function that results in drug addiction. Epigenetic changes and potential structural adaptations regulate the manifestations of METH use disorder (MUD) in humans. METH addiction is accompanied by significant changes in gene and protein expression related to epigenetic mechanisms within specific brain areas. These findings suggest that epigenetics is connected with METH addiction.

The behavioral effects of addictive drugs are examined by the conditioned place preference (CPP) and drug self-administration procedures in epigenetic studies (Hamilton and Nestler, 2019; Werner et al., 2021). Injection of sodium butyrate (NaBut), a non-specific histone deacetylase inhibitor, immediately after administration promotes extinction of METH-CPP and reduces METH-CPP reinstatement (Werner et al., 2021).

### Histone acetylation and methamphetamine addiction

Histone acetylation is most studied in chromatin modification in animal models of addiction. The acetylation of histones, histones deacetylases (HDACs), histone acetyltransferases (HATs), histones methyltransferases (HMTs/KMTs), histones demethylases (HDMs/KDMs) are related with METH addiction.

The acetylation of histones3 (H3) and H4 were increased, particularly H3 in the PFC when METH induced behavioral sensitization (Li H et al., 2021). The alterations in transcription and histone acetylation induced by METH in the PFC in rats are involved in behavioral sensitization induced by METH (De Sa Nogueira et al., 2019). METH increased the expressions of H2BAc, H3K9Ac, H4K12Ac in the Hip, and H4K12Ac expression in the striatum (Godino et al., 2015). Repeated METH treatment increased H4AC enrichment at D1, hypocretin (orexin) receptor 1 (HCRTR1), and N-Methyl-d-Aspartate receptor 1 (GRIN1) promoters but less enrichment of H3AC at the promoters of D2, HCRTR1, HCRTR2, histamine receptor H1(HRH1), HRH3, and NNMDA in the mPFC (Gonzalez et al., 2018).

Histone deacetylases (HDACs) are expressed in various brain areas and regulate gene expression induced by drugs. There are four classes of HDACs (I, II, III, and IV), a single administration of METH can affect their expressions in the NAc (Gonzalez et al., 2019). A single treatment of METH reduces the mRNA level of HDAC3 in the NAc (Torres et al., 2016). However, a large single dose of METH (20 mg/kg) decreases HDAC1 expression, but increases HDAC2 expression in the NAc in rats (Nestler and Luscher, 2019). HDAC2 conditional knockdown extends the expression of immediate early genes in the NAc in mice after acute METH exposure (Torres et al., 2015). A single METH treatment reduces mRNA level of HDAC8 in the NAc in mice. Similar, HDAC11 mRNA level is decreased after acute METH exposure in the NAc in mice (Torres et al., 2016).

METH impacts the PFC and results in cognitive decline and addiction. METH regulates gene expression based on its effects on histone markers at gene promoter. METH causes differential alterations in gene expression and histone acetylation in the mPFC in rats. METH induces histone acetylation and increases HDAC1, HDAC2 protein levels in the mPFC in mice. H3 acetylation is significantly enriched at the promoter of D1R (Li H et al., 2021).

METH induced activation of D1 receptor (D1R) in the PFC, which affected HDACI and HDACII levels, miRNA 181a/d level in the VTA, increased the expressions of *a*-adrenergic receptors and N-methyl-d-aspartate (NMDA) receptor subunit, then regulated the function of dopamine receptors (DRs) (Gonzalez et al., 2019; Jayanthi et al., 2021). However, repeated METH treatment did not change the mRNA level of HDAC3 in the mPFC (Gonzalez et al., 2020). A single METH treatment increased mRNA level of HDAC8 in the mPFC in mice (Gonzalez et al., 2020). Moreover, decreased HDAC2, HDAC8, and HDAC9 mRNA levels in the DS enhanced rats sensitivity to METH (Cadet et al., 2019). Fos-positive neurons increased HDAC3 mRNA level after withdrawal from METH self-administered (SA) in the DS in rats (Li et al., 2015). HDAC5 overexpression in the DS increased METH seeking after withdrawal. In contrast, HDAC5 knockdown in the DS decreased METH seeking after withdrawal, which suggests that HDAC5 expression in the DS is involved in incubation of METH craving (Li X et al., 2018). These findings identified that HDAC5 was a target for mediating METH addiction. Other investigators used METH SA in rats to assess the role of HDACs in drug taking behavior. They found that HDAC5 overexpression in the DS by using viral vectors increased METH-seeking behavior, but HDAC5 knockdown decreased METH-seeking behavior. METH induced an increase in HDAC5 level in the DS, and knockdown of HDAC5 in the DS suppressed D1 and D2 expressions (Li X et al., 2018). High concentration of HDACs inhibitors increased histone H3 acetylation by inhibiting HDAC1 activity, and low concentration of inhibitors increased *a*-tubulin acetylation by inhibiting HDAC6. Through the above regulation, HDACs inhibitors reversed neuronal morphological changes induced by METH in human neuroblastoma SH-SY5Y (Sharma et al., 2019). The number of active lever presses in METH self-administration rats was reduced by pretreatment with an inhibitor of HDAC6. METH administration increased GluN2B, an NMDA receptor subunit expression and sequential activation of extracellular regulated protein kinases (ERK)/cAMP-response element binding protein (CREB)/brain derived neurotrophic factor (BDNF) pathway which were abolished by an inhibitor of HDAC6 in the Hip, which suggests that HDAC6 inhibitor prevents METH self-administration in rats (Kim et al., 2020). Collectively, HDAC6-isoform selective inhibitor provided therapeutic potential with the treatment of METH addiction. METH decreased the mRNA level of HDAC8 in the DS in mice (Omonijo et al., 2014). Repeated METH treatment reduced mRNA levels of HDAC10 and HDAC11 in the DS in rat (Omonijo et al., 2014; Cadet et al., 2019).

Histone acetyltransferases (HATs) are also involved in METH addiction. ATF-2, a member of the ATF/CREB family, has the intrinsic HAT activity on histone H4 and increases CRE-dependent transcription (Martin et al., 2012). P300, one kind of HATs is increased by METH in human primary astrocytes which play a role in regulating Glu release induced by METH (Doke et al., 2021).

Histone methylation is a novel molecular mechanism that can also influence METH-induced behavioral sensitivity. However, histone methylation has not been well studied in addiction models. Trimethylation of histone H3 at lysine 4 (H3K4me3) was found to be increased in the NAc in models of METH-induced behavioral sensitization (Ikegami et al., 2010). Methyltransferases (HMTs/KMTs) and demethylases (HDMs/KDMs) were involved in METH addiction, such as KMT2A, an enzyme involved in H3K4me3, was increased by METH and required for METH-associated memory formation and maintenance. KDM5C demethylates H3K4 and was associated with METH-CPP (Aguilar-Valles et al., 2014). The NAc plays the most important role in METH priming-induced CPP reinstatement. Some studies reported that decreased mixed lineage leukemia 1 (Mll1), a histone methyltransferase (HMT) or KDM5C (a histone lysine demethylase) expressions by using siRNAs in the NAc reduced METH-induced CPP expression (Werner et al., 2021).

From the above studies, it is suggested that histone acetylation plays the regulating roles in METH addiction. Histone acetylation modification is related to histone acetylation level, the expressions of HDACs, HATs, histone methyltransferase and histone lysine demethylase. Moreover, neurotransmitters, the receptors and the signaling pathways are altered by histone acetylation after METH treatment. The modifications are included in Table 1. It is showed that the modifications are different in the specific brain areas related to METH addiction. The differences in these findings could be due to several factors, including the METH (time and dose), self-administration procedure or CPP, brain regions (PFC, mPFC, HiP, DS, NAc) and the behaviors (seeking, craving, relapse, the CPP expression, consolidation, extinction, reinstatement and locomotor sensitization). Thus, histone acetylation consequences at a given location could in and of themselves mediate the targets in fully different neurons (DA, Glu, GABA). Researches will isolate the histone acetylation modifications that drive the most important elements of METH addiction.

**TABLE 1:** The epigenetics molecules, brain areas, targets in METH addiction.

Epigenetic	Molecules↓↑	Brain areas	Target	Citation
Acetylation of histones	H3↑, H4↑, H2BAc↑, H3K9Ac↑, H4K12Ac↑	DS, Hip, PFC	D1↑, D2↑, HCRTR1↓↑,HCRTR2↑, HRH1, 3↑, NMDA↑	Li H et al. (2021)
				Godino et al. (2015)
				Gonzalez et al. (2018)
HDACs	HDAC1↑↓, 2↑↓, 3↓, 5↑, 6↑, 8↑↓, 9↓, 10↓, 11↓	DS, Hip, NAc, PFC, mPFC	immediate early genes↓, *a*-adrenergic receptors↑, BDNF↑, NMDA↑, D1↑, D2↓, CREB↓, *a*-tubulin↑	Torres et al. (2016)
				Nestler and Luscher, (2019)
				Li H et al. (2021)
				Cadet et al. (2019)
				Gonzalez et al. (2020)
				Sharma et al. (2019)
				Kim et al. (2020)
				Omonijo et al. (2014)
HATs	ATF-2↑, p300↑	DS, Hip, PFC, mPFC	CRE↑, Glu↑	Martin et al. (2012)
				Doke et al. (2021)
Histone methylation and DNA methylation	KDM5C↑, KMT2A↑, LINE↑, DNMT↑↓, MeCP↑↓, Shati/NAT8L↑, Mll1↑, HMT↑	DS, Hip, NAc, PFC, mPFC	BDNF↑, OT↓, K^+^ channel↑↓, Syp↑↓, *a*-Syn↑, Glu↑, GluA1,2↑, LINE-1↑, NR4A1, GABA↓	Ikegami et al. (2010)
				Aguilar-Valles et al. (2014)
				Werner et al. (2021)
				Miller et al. (2008)
				Iamjan et al. (2021)
				Salehzadeh et al. (2020)
				Moszczynska et al. (2015)
				Walker and Nestler, (2018)
				Cadet et al. (2019)
				Fan et al. (2020)
				Biagioni et al. (2019)
				Yuka et al. (2020)
miRNAs	miRNA128↑, 237↑, 296↑, 501↑ 31–3p↑, 34a-5p↑, 183–5p↑, 9a-5p↑, 369–3p↑, 29a↑, 181a/d↑	DS, Hip, NAc, VTA	PKG↑, PI3K↑, Wnt↑, Ago2↑, NRG-1↑, BDNF↑, GluN1↑	Li H et al. (2018)
				Li J et al. (2021)
				Yang et al. (2020)
				Liu et al. (2021)
				Zhou et al. (2021)
				Qian et al. (2021)
				Chand et al. (2021)
				Kim et al. (2022)
LncRNAs	Kcnq1ot1↑, Zfhx2as↑, Neat1↑, Neat2↑, Miat↑	Hip, NAc	Camk4↑,CREB1↑, AMPA α1↑, CREB-binding protein↑	Kapranov et al. (2007)
				Zhu et al. (2015)
Ubiquitination	Parkin↑, SYVN1↓	BLA, CeA, DS	D1, 2↑, NMDA↑, AMPA↑, GABAAα1↑	Sharma et al. (2021)
				Jiao et al. (2017)
				Alonso and Friedman, (2013)
				Peeler et al. (2017)

### Methylation and methamphetamine addiction

DNA methylation at gene promoters is usually used to study variation in methylation status. Methyl groups are added to the 5′ positions of the pyrimidine rings of cytosine residues located in CpG dinucleotide islands to regulate gene expression. This addition of methyl groups to cytosines in the DNA is regulated by the activity of DNA methyltransferases. DNA demethylation is catalyzed by DNA methyltransferase (DNMT). DNA demethylation induces synaptic plasticity in the Hip and governs the neuroplasticity regulated by psychostimulants (Miller et al., 2008). The genome-wide DNA methylation assays have shown the alterations in DNA methylation status in blood of drug addiction patients (Cecil et al., 2016). It has been reported that DNA methylation level is altered in METH addicted patients (Liu et al., 2020) and in the children of METH addicted parent (Asaoka et al., 2020). There was hypermethylated or hypomethylated CpG in brain tissues of drug addiction patients. In addition, some gene methylation was found in the candidate genes involved in drug addiction and other psychiatric disorders (Papageorgiou et al., 2019; Asaoka et al., 2020). BDNF methylation was increased in the PFC of METH-addicted rats and patients, but was decreased in the Hip of rats (Iamjan et al., 2021). The decrease of BDNF expression contributed to the neurotoxic effects of METH exposure (Salehzadeh et al., 2020; Iamjan et al., 2021). The single dose of METH increased striatal DNA (cytosine-5) methyltransferase 1 (DNMT1) mRNA in rats (Jayanthi et al., 2018). Different paradigms of METH induced DNA methylation in the methylated DNA immunoprecipitation (MeDIP) (Jayanthi et al., 2014; Jayanthi et al., 2020). The long-interspersed element-1 (LINE-1) in the DNA is known to cause genome instability, which is regulated by DNA methylation and histone modifications. According to the report by Moszczynska et al., when METH induced impairment in cognition and memory, the activity of LINEs in the dentate gyrus of Hip and the DS was increased (Moszczynska et al., 2015).

Recent research showed that the changes in DNA methylation and mRNA expression of potassium channels were blocked in the rat NAc and METH self-administration was enhanced by a single prior injection of METH (Cadet et al., 2019; Jayanthi et al., 2020). METH downregulated the expressions of DNMTs and methyl CpG binding protein 2 (MeCP2), reduced DNA methylation at Synaptophysin (Syp) promoter in the Hip and enhanced spatial memory. Whereas METH upregulated the expressions of DNMTs and MeCP2, induced DNA hypermethylation at Syp promoter in the PFC and impaired cognitive memory. Specific knockout of MeCP2 in the NAc enhanced the rewarding effect of amphetamine (Walker and Nestler, 2018). Oxytocin (OT) inhibited METH-seeking behaviour and relapse by reversing DNA methylation at Syp promoter, regulating the expressions of DNMTs and MeCP2 in the Hip and PFC (Fan et al., 2020). The exogenous OT reduced the reinstatement of METH-seeking behavior by increasing the inhibitory signal in the prelimbic cortex (PrL) to reduce the output of Glu to the NAc induced by METH (Everett et al., 2019). Long term administration of METH inhibited CpG demethylation at SNCA promoter and increased *a*-synuclein (α-Syn) expression in the striatal neurons. The demethylation persists even during METH withdrawal periods (Biagioni et al., 2019). The PFC is central to the neural circuitry underlying memory extinction, and modulation of the mPFC influences extinction and subsequent relapse of drug memories (Zhang W.H et al., 2019). Studies have demonstrated that METH DNA methylation at CpG islands of SHATI/NAT8L is increased in METH users (Yuka et al., 2020). METH-CPP is inhibited by Shati/Nat8l overexpression in the mPFC (Haddar et al., 2020). Several CpG sites of the Arc and the Fos have significant changes in DNA methylation status in the PFC of chronic METH-treated mice, while the Krueppel-like factor 10 (KLF10) and the Orphan nuclear receptor NR4A1 have significant changes in the Hip (Cheng et al., 2015). The parvalbumin (PV)-containing subgroup of GABAergic neurons is particularly affected in schizophrenia and animal models of psychosis, including after METH administration, parvalbumin (PVALB) methylation is increased in METH dependence and METH-induced psychosis (Veerasakul et al., 2017). METH decreased the enrichment of 5-methylcytosine and 5-hydroxymethylcytosine at GluA1 and GluA2 promoter sequences (Jayanthi et al., 2014).

The above researches suggests that in different brain areas DNA methylation mediates long-lasting changes at promoters of genes related to METH abuse.

### Noncoding RNAs and methamphetamine addiction

MicroRNAs (miRNAs) are one of non-coding RNAs, which do not translate into proteins but still perform the crucial roles in transcription and post-transcriptional events (Zhang P et al., 2019). Potential involvement of miRNAs has been fully examined in METH addiction. The potential roles may also be treatment options for METH addiction. MiRNAs work as the regulators of genes involved in METH-mediated changes in dendritic spines and synaptic transmission (Li H et al., 2021; Li J et al., 2021; Qian et al., 2021; Wang et al., 2021). METH-CPP is accompanied by the upregulation and downregulation of miRNAs in serum exosomes (Wang et al., 2021). These miRNAs-regulated genes have been shown to be involved in vesicular transport, amphetamine addiction, cyclic guanosine monophosphate cGMP-protein kinases G (PKG) signaling pathway, dopaminergic synapse, and GABAergic synapse by using the KEGG pathway analysis (Li H et al., 2018). In the central amygdala and orbitofrontal cortex, the expressions of multiple miRNAs are increased together with molecules related to METH addiction in genome wide transcriptional profiling (Cates et al., 2018). When METH induced CPP expression, levels of miRNAs 237, 296, and 501 in the NAc were increased. The miRNAs in the NAc regulate genes involved in Wnt signaling and axon guidance (Yang et al., 2020). MiR-128 influenced METH-induced behavioral sensitization through changing the molecules related to synaptic plasticity in the NAc (Li J et al., 2021). Ago2-dependent miRNAs in the NAc disrupt METH-induced locomotor sensitization. These effects of Ago2/miR-3068-5p happen together with the glutamate receptor, GluN1/Grin1 (Liu et al., 2021). METH induced miRNAs expressions in the striatum, damaged motor coordination, reduced striatal volume and dendritic length (Chavoshi et al., 2020). High-throughput sequencing analysis showed that miRNAs expressions were dysregulated by METH, 113 up-regulated and 54 down-regulated in the DS in rats. The changes were involved in phosphatidylinositol 3′-kinase (PI3K)-Protein kinase B (Akt) and FoxO signaling pathway (Chavoshi et al., 2020). METH might impact the expressions of miRNAs in Extracellular Vesicle (EV) (Sandau et al., 2020; Chivero et al., 2021). When CPP expression was induced, EV-containing miRNAs (miR-183-5p, miR-9a-5p, and miR-369-3p) were increased in the Hip in mice (Zhou et al., 2021). MiR-183-5p inhibited METH addiction by regulating NRG-1 expression in mice (Zhou et al., 2021). In the dorsal Hip (dHIP), miRNA-31-3p/RhoA pathway was involved in METH-CPP (Qian et al., 2021). Studies in humans with METH addiction have documented changes in plasma extracellular vesicles (EV). MiR-137 in the circulating EV is a stable and accurate diagnostic marker of METH abstinence syndrome (Kim et al., 2022). EV-associated miR-29a plays a role in METH-induced inflammation and synaptodendritic damage. Whereas treatment with the anti-inflammatory drug, ibudilast (AV411), which is known to reduce METH relapse, decreased the level of miR-29a, subsequently attenuated inflammation and rescued synaptodendritic injury (Chand et al., 2021). MiR-29a level was increased in drug-seeking and reinstatement in a rat METH self-administration model. Brain-derived EV (BDE) miRNA and miR-29a-3p (mir-29a) were significantly increased during chronic METH exposure. Sandau et al. reported different expressions of miRNAs, 19 up-regulated and 69 down-regulated in the peripheral blood of female abusers by using a miRNA array platform. Interestingly, age of first use and lifetime use of METH were also related to miR-628-5p expression, miR-301a-3p and miR-382-5p (Sandau et al., 2020).

LncRNAs are longer than 200 nt that do not encode protein (Kapranov et al., 2007). As epigenetic regulators, lncRNAs have been found to be involved in METH addiction. Zhu et al. found that METH induced global changes in lncRNAs expressions in the NAc of sensitized mice through high throughput ssRNA-seq technology. In their study METH regulated five lncRNAs (Kcnq1ot1, Zfhx2as, Neat1, Neat2, and Miat) and the corresponding protein-coding genes, such as, calcium/calmodulin dependent protein kinase IV (CaMk4), CREB1, CREB-binding protein, Glu receptor, ionotropic, AMPAα1 and mitogen-activated protein kinase10 (MAPK10) which involved in synaptic transmission (Zhu et al., 2015). These lncRNAs regulated the expressions of their sense partners which have been reported to be involved in the modulation of LTP in the Hip and neuronal plasticity (Bernard et al., 2010).

Evidence from numerous studies has demonstrated that different miRNAs in the different brain areas regulate METH addiction in distinct ways.

### Ubiquitination and methamphetamine addiction

Ubiquitin Proteasome System (UPS) is a multi-enzyme system which regulates proteolysis and turnover. It has been reported that UPS is involved in neurotransmitter transmission and synaptic plasticity in DA-related brain disorders. Pre- and post-synaptic neurons in the DA circuitry are sensitive to UPS inhibition. The UPS decreases both D1/D2-like DRs, and Alpha-amino-3-hydroxy-5-methyl-4-isoxazolepropionic acid (AMPA) through endocytic internalization and degradation (Alonso and Friedman, 2013; Peeler et al., 2017). DA transmission is controlled by the UPS, which regulates the presynaptic release of Glu via both D1-like and D2-like receptors (Briones-Lizardi et al., 2019). The UPS also modulates synaptic plasticity at post-synaptic sites (Dong et al., 2014; Hegde, 2017). Thus, DA and Glu signaling pathways are interacted with UPS substrates (Bach and Hegde, 2016). UPS activity and dopaminergic transmission are regulated by METH (Limanaqi et al., 2019). Parkin increases the ubiquitination of substrate proteins to enhance their degradation. Parkin activity is associated with METH addiction in rats (Sharma et al., 2021). Protein ubiquitination and E3 ubiquitin ligases are increased in the central amygdala (CeA) after METH withdrawal. The ubiquitination in the CeA is also closely related to METH craving behavior (Cates et al., 2018).

Synovial apoptosis inhibitor 1 (SYVN1) is an endoplasmic reticulum (ER)-associated degradation (ERAD) E3 ubiquitin ligase. It has been reported that SYVN1 knockdown is related to METH-CPP by increasing GABA_A_α1 in the DS (Jiao et al., 2017). MiRNA-181a also regulates METH addiction through the ERAD pathway (Wang et al., 2021). These studies provide evidence that the UPS is linked in METH addiction.

## Conclusion

METH addiction is related to neurotransmitters of DA, Glu, NE and serotonin in the mPFC, the VTA and the NAc. The neurotransmitters are regulated by histone acetylation, methylation, miRNAs and ubiquitination in the brain areas. Actually, these epigenetic mechanisms do not alone regulate addiction induced by METH, they work together, such as miRNAs regulation on UPS (Wang et al., 2021). The epigenetics related to molecules and their targets involved in METH addiction are concluded in Table 1. The epigenetic mechanisms underlying the behavioral effects of addictive drugs need to be studied further.
